# Electroacupuncture pretreatment protects against anesthesia/surgery-induced cognitive decline by activating CREB via the ERK/MAPK pathway in the hippocampal CA1 region in aged rats

**DOI:** 10.18632/aging.205124

**Published:** 2023-10-19

**Authors:** Hongjie Huang, Yanan Li, Xupeng Wang, Qi Zhang, Juan Zhao, Qiujun Wang

**Affiliations:** 1 Department of Anesthesiology, The Third Hospital of Hebei Medical University, Hebei 050051, China; 2 Department of Anesthesiology, Hebei Children’s Hospital Affiliated to Hebei Medical University, Hebei 050031, China; 3 Experimental Teaching Center, Hebei Medical University, Hebei 050011, China

**Keywords:** electroacupuncture, postoperative cognitive dysfunction, synaptic damage, aged, ERK/MAPK signal pathway

## Abstract

Effective preventive measures against postoperative cognitive dysfunction in older adults are urgently needed. In this study, we investigated the effect of electroacupuncture (EA) on anesthesia and surgery-induced cognitive decline in aged rats by RNA-seq analysis, behavioral testing, Golgi-Cox staining, dendritic spine analysis, immunofluorescence assay and western blot analysis. EA ameliorated anesthesia and surgery induced-cognitive decline. RNA-seq analysis identified numerous differentially-expressed genes, including 353 upregulated genes and 563 downregulated genes, after pretreatment with EA in aged rats with postoperative cognitive dysfunction. To examine the role of CREB in EA, we injected adeno-associated virus (AAV) into the CA1 region of the hippocampus bilaterally into the aged rats to downregulate the transcription factor. EA improved synaptic plasticity, structurally and functionally, by activating the MAPK/ERK/CREB signaling pathway in aged rats. Together, our findings suggest that EA protects against anesthesia and surgery-induced cognitive decline in aged rats by activating the MAPK/ERK/CREB signaling pathway and enhancing hippocampal synaptic plasticity.

## INTRODUCTION

Postoperative cognitive dysfunction (POCD) is a severe complication of surgery and anesthesia in elderly patients that manifests as acute delirium and long-term neurocognitive decline, including mental disorders, anxiety, personality changes and memory impairment [1]. Because of POCD, aged patients are at higher risk of permanent cognitive impairment and dementia, and are more likely to die after surgery, incurring a severe psychological and financial burden on their families [2]. Over the past decade, an increasing number of aged patients have undergone general anesthesia surgery because of the aging population and improvements in surgery and anesthesia technology [3, 4], resulting in an increased incidence of POCD.

The mechanisms underlying POCD are unclear, but numerous studies show that central inflammation [5], oxidative stress [6], apoptosis [7] and synaptic plasticity changes [8] in the hippocampus contribute to POCD following anesthesia/surgery. Dysregulated synaptic plasticity is associated with cognitive dysfunction [9–11]. Gao reported that POCD in aged mice having undergone laparotomy under anesthesia was related to a decrease in the synaptic plasticity protein PSD-95, as well as synaptic morphological changes in the hippocampus [9].

Clinically and experimentally, electroacupuncture (EA) exerts preventive effects against cognitive dysfunction in POCD models by controlling stimulation frequency and intensity [12, 13]. Our group has previously found that EA improves cognitive decline following spine surgery in elderly patients [14]. Additionally, we have shown that pretreatment with EA improves anesthesia-induced cognitive dysfunction via inhibition of mitochondrial injury and apoptosis in aged rats [15, 16]. There is, however, no clear mechanism underlying EA’s ability to prevent POCD in elderly. It has been reported that EA improves hippocampal synaptic plasticity and exerts neuroprotective effects against cerebral ischemia-reperfusion injury [17]. In addition, ERK/MAPK pathway is a key pathway regulating synaptic plasticity [18]. Interestingly, EA has also been reported to inhibit astrocyte activity in the spinal cord dorsal horn of rat with IBS visceral hypersensitivity by inhibiting ERK/MAPK pathway [19]. Gao HY showed EA can achieve analgesic effects by regulating amino acid metabolism and activating the MAPK signaling pathway [20]. Currently, the role of ERK/MAPK pathway in EA against AS-induced POCD is unclear.

Here, we investigate the mechanisms underlying the neuroprotective effects of EA on anesthesia/surgery-induced cognitive decline in aged rats, with the aim of identifying potential therapeutic and preventive targets for POCD.

## MATERIALS AND METHODS

The animal protocols complied with the Chinese Guidelines for the Care and Use of Laboratory Animals. Each experimenter obtained a certificate of training as a laboratory animal practitioner. Efforts were made to reduce the number of animals used. Animal protocols were approved by the Animal Review Board of the Third Hospital of Hebei Medical University (Ethical code: KSD2023-049-1).

### Animals and grouping

Healthy male Sprague-Dawley rats, 20 months of age and weighing 550–650 g, were obtained from the Experimental Animal Center of Hebei Medical University (Permit No. SCXK 2022-001). The number of rats needed to generate statistically significant differences was calculated using standard power calculations. Power and sample sizes were calculated using an online tool (http://www.stat.uiowa.edu/~rlenth/Power/index.html) for the various tests, based on variability of the assays and inter-individual differences within groups. The rats were kept in a room with a temperature of 25°C, humidity of 55%, and a 12-h/12-h light/dark cycle. In the daytime, animals were randomly grouped and subjected to double-blind experiments.

**Figure 1:**
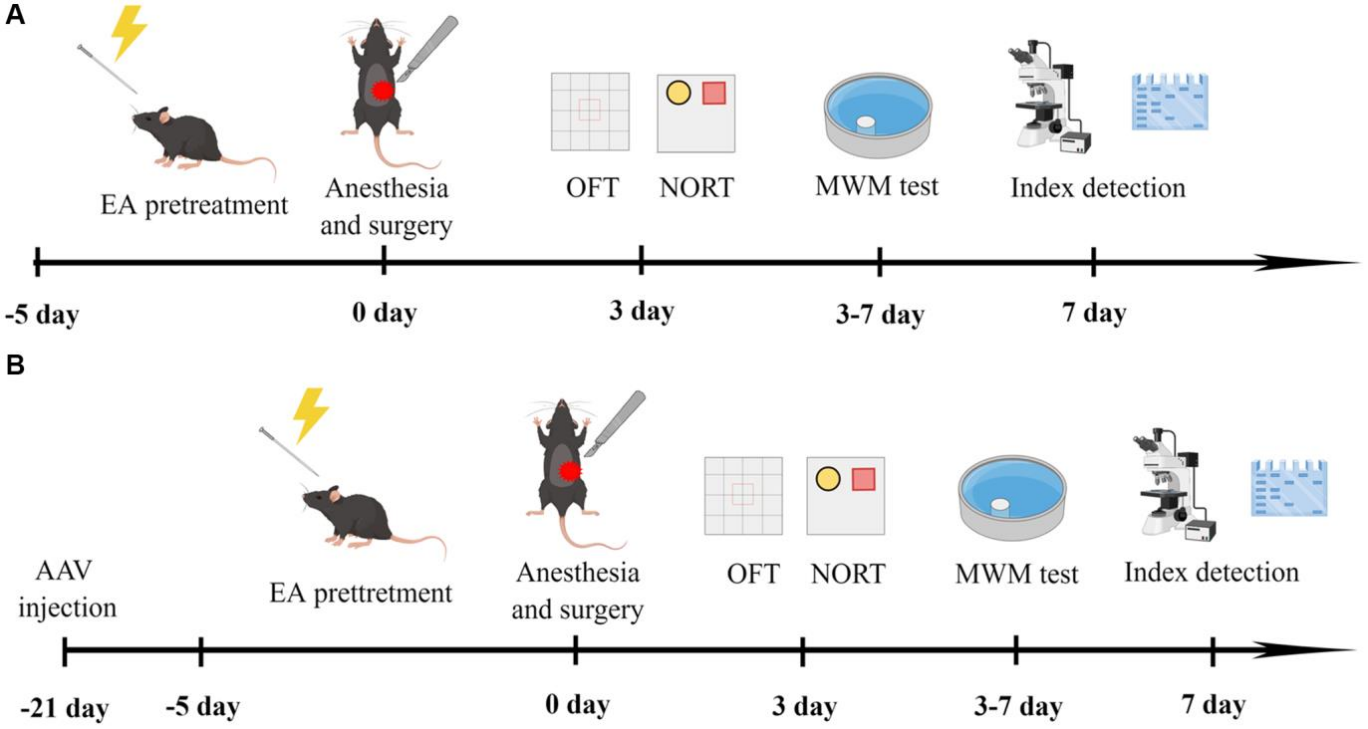
**Experimental flow chart of the study (drawn by Figdraw, ID: WYUTPfff42).** (**A**) Rats were treated with EA for 5 days, and then subjected to anesthesia and exploratory laparotomy 24 h after the final EA treatment. On the 3rd day after fracture surgery, mobility of aged rats was assessed with the open-field test (OFT) and cognitive function was evaluated with the novel object recognition test (NORT). On days 3–7 after surgery, cognitive function was assessed with the MWM test. (**B**) Twenty-one days before surgery, rats received bilateral stereotaxic injections of AAV-shCREB into the CA1 region. Rats were subjected to anesthesia and exploratory laparotomy 24 h after the final EA treatment. On the 3rd day after fracture surgery, mobility was evaluated with the OFT and cognitive function was assessed with the NORT. On days 3–7 after surgery, cognitive function was assessed with the MWM test.

In the first experiment, the aged rats were divided into four groups: C (*n* = 12), C+EA (*n* = 12), POCD (*n* = 19) and POCD+EA (*n* = 19). Mice in group C did not receive anesthesia and surgical stimulation. Rats in group POCD and POCD+EA received anesthesia and surgical stimulation. Rats in group C+EA and POCD+EA received EA stimulation before anesthesia and surgery. The schematic diagram of the experimental procedure is shown in Figure 1A.

### Anesthesia/surgery model establishment

The POCD model was established using exploratory laparotomy under sevoflurane anesthesia, as described in the previous study [21]. Anesthesia was induced with 7.0% sevoflurane and maintained with 3.0% sevoflurane. After the abdomen was disinfected, an incision (approximately 3 cm) was made vertically on medial line of the abdomen, followed by detection of organs, including the liver, kidney, spleen and the intestines. After confirmation of absence of bleeding, the wound was closed using sterile sutures, and the rats were kept at 37°C for regaining consciousness. After rats recovered from anesthesia/surgery, lidocaine (2%) was applied locally to treat postoperative pain.

### EA pretreatment

According to the method described previously [15], rats in C+EA and POCD+EA received a 5-day persistent EA treatment at Neiguan acupoint (PC6, situated at a distance of approximately 3 mm from the transverse stripe of the wrist, specifically at the axopetal end), Hegu acupoint (LI4, situated between the first and second metacarpal bones) and Zusanli acupoint (ST36, located at 5 mm under fibular head of the hind limb), as indicated in the textbook for experimental acupuncture. The rats received fixation and needled at Baihui, Dazhui and Zusanli acupoints with a needle depth of 2–3 mm from the vertical angle using the stainless steel needles (0.35 mm × 13 mm). All needle ends were connected to EA stimulator instrument separately (Model G6805; SMIF, Shanghai, China). Rat whiskers slight jitter is the effective sign for EA at Baihui and Dazhui, and the slight shaking of hind limbs in rats is an effective marker for EA stimulation of Zusanli. The EA parameters set included sparse-dense wave (2/15 Hz), intensity 1 mA, continuous stimulation of dilatational wave. The rats in other groups don’t receive any management.

### RNA isolation and sequencing

RNA sequencing was performed between POCD and POCD+EA group. The hippocampal CA1 region of 7 rats were isolated on ice 7 days after surgery, and total RNA was isolated and purified using TRIzol (Invitrogen, Carlsbad, CA, USA) according to the manufacturer’s protocol. A NanoDrop ND-1000 (NanoDrop, Wilmington, DE, USA) was used to quantify RNA amount and purity. An Agilent 2100 (Thermo Fisher Scientific, Waltham, MA, USA) was used to assess RNA integrity, and RNA with a RIN of >7.0 was used.

RNA (poly(A)) was purified from total RNA (5 μg) using poly-oligo-T-conjugated magnetic beads (2×) and fragmented using divalent cations at high temperature. The RNA was reverse-transcribed into cDNA, and an adenine nucleotide was added to the blunt ends of each strand, followed by ligation to Illumina multiplex barcode adapters. Amplification noise and sequence-dependent bias were minimized with custom Unique Molecular Identifiers based on Shiroguchi’s work [22], and AMPureXP beads were used for size selection. The following PCR conditions were used: initial denaturation at 95°C for 3 min; 8 cycles of denaturation at 98°C for 15 s, annealing at 60°C for 15 s, and extension at 72°C for 30 s; final extension at 72°C for 5 min. Final cDNA library insert size was 300 (50) bp on average. Sequencing was carried out on the Illumina HiSeq 4000 (LC Bio, Hangzhou, China) according to the recommended protocol. Using DEseq, we identified differentially expressed genes (DEGs) and significant genes with fold changes of >2, with an adjusted *p*-value of 0.05.

### Functional enrichment analysis

Database for Annotation, Visualization and Integrated Discovery (DAVID) (http://david.ncifcrf.gov/) online database tools were used to create DEGs for biological processes. A ggplot2 package was used to map the GO pathways of DEGs using R. GSEA was used for enrichment analyses of all genes, and GSEA was used to map pathways [23, 24].

### Viral plasmids

We generated AAV plasmids to down-regulated CREB according to the above method. We generated shRNAs targeting endogenous CREB using an adeno-associated viral shRNA expression system. CREB-targeting sequences were cloned into the pAAV-ZsGreen-shRNA (Item No. P1619, Miaoling Biology, Wuhan, China) between *BamHI* and *Hind III* following PCR primers: 5′-CGGGATCCGCCATCAGTTATCCAGTCTCCTTCAAGAGAGGAGACTGGATAACTGATGGCTTTTTG-3′ and 5′-CAAGCTTGCCATCAGTTATCCAGTCTCCTCTCTTGAAGGAGACTGGATAACTGATGGC-3′.

### Stereotactic injection of virus

In the following experiment, mice were divided into three groups (*n* = 12): POCD+AAV-ZsGreen, POCD+AAV-ZsGreen+EA and POCD+EA+AAV-shCREB respectively. The schematic diagram of the experimental procedure is shown in Figure 1B.

At 21 days before anesthesia and surgery, anesthesia induction chambers prefilled with 5% sevoflurane were used to induce anesthesia, and a stereotactic apparatus with a single arm was used to fix the mice when their reflexes disappeared. The skin was then prepared, disinfected, and 0.5% lidocaine was locally applied to the scalp. After calibrating the skull, small holes were drilled 1.8 mm posterior and 1.2 mm lateral to the bregma. Glass electrodes were inserted into the hippocampus at a depth of 1.8 mm to reach the CA1 region. An injection of 500 nl pAAV-CMV-ZsGreen-shCreb1 (AAV-shCREB) was made at 50 nl/min. The needle was maintained in place for 10 min after injection, and then slowly removed. The incision was sutured and covered with bone wax.

### Behavioral tests

A wide range of behavioral tests were conducted, including the Morris water maze (MWM) test, the novel object recognition test (NORT) and the open-field test (OFT). On day 3 after surgery, the OFT was conducted to exclude an impact of anesthesia/surgery-induced changes in spontaneous locomotor activity on cognitive function test results [25]. A box consisting of plexiglass walls and a black floor was used as an open-field apparatus. After acclimating, mice were placed in the center of the box and allowed to roam freely for 5 min. EthoVision™ XT software (Noldus Information Technology, Wageningen, The Netherlands) was used to analyze distance, speed and duration of open-road travel using overhead video cameras.

Aged rats were evaluated for cognitive ability 1 h after OFT using the NORT, as described previously [25]. The NORT consists of the following three phases: adaptation to the environment, familiarization with the object, and recognition of the novel object. After the adaptation phase, rats explored freely for 5 min in a black box (60 × 60 × 40 cm). The rats were placed in the center of two identical cube objects (A and B) inside the experimental box and allowed to explore both objects. The time spent exploring each object was recorded. Video analysis software (XR-XZ301, Xinruan, Shanghai, China) was used to record the exploration times of objects A and C over a 5-min period after the right cube object B was removed and replaced with cylindrical object C. The recognition index (time spent exploring novel objects/time spent exploring familiar objects + novel objects) was used to measure cognitive ability.

The MWM test was conducted on days 3 to 7 after anesthesia/surgery to assess memory and learning abilities [15]. The circular pool (150 cm in diameter, 50 cm in height) was filled with water (24–26°C) to a depth of 32 cm. To make the water opaque, nontoxic black ink was added, and the pool was divided into four quadrants (I, II, III and IV). Two centimeters below the water surface, a circular platform was located in quadrant IV with a diameter of 6 cm and height of 30 cm [26]. Reference cues were placed around the pool, as well as the lights in the room. The MWM test consisted of a 4-day training phase and a 1-day test phase. During the training phase, a total of four starting positions were used in the experiment, and if the mouse failed to board the platform within 120 s, it was guided onto it for 15 s. Following the training session, the pool was cleaned every day to eliminate odors. Training sessions were conducted four times a day. During the test phase, escape latency was measured (i.e., the time from release to boarding the platform). Upon crossing the original platform area, the total time spent in the target quadrant after the platform had been removed was recorded over a 60-s period. The MWM test was conducted using the JLBehv-MWM system (Shanghai Ji’Liang Software Technology Co., Ltd., Shanghai, China).

### Golgi-cox staining

Rats were given deep anesthesia with 7% sevoflurane 1 h after the MWM test (*n* = 3). The left hemisphere was removed and fixed in 4% paraformaldehyde for 48 h. A 2-mm-thick piece of brain tissue was cut, gently rinsed several times with saline, and then stained with Golgi solution, which was changed every 3 days for 14 days in a cool, ventilated environment. Following three washes with double distilled water (ddH_2_O), the brain tissue was incubated overnight in 80% glacial acetic acid. After rinsing in ddH_2_O and dehydration in 30% sucrose, the brain tissue was cut into 100-μm sections using an oscillating microtome and attached to gelatin slides. After air-drying the slides, concentrated ammonia was injected for 15 min, followed by 1 min of rinsing in ddH_2_O. Finally, slides were incubated for 15 min in acidic film fixative, rinsed with ddH_2_O for 3 min, air dried, and sealed with glycerol gelatin. Image-Pro Plus 6.0 software (Media Cybernetics, Rockville, MD, USA) was used to quantitatively analyze two microscopic fields for each mouse in the hippocampus bilaterally.

Dendritic spines of the second or third branch of the neuron were measured in the 30–90 μm length range. Using the following formula, we calculated the density of dendritic spines per 10 μm: Dendritic spine density = number of spines/dendritic length × 10. The Sholl analysis plug-in was used to draw 10 concentric circles with 10-μm spacing from the cell body, and the number of intersections between the dendrites and the concentric circles was calculated.

### Long-term potentiation (LTP) recording

Following MWM testing, rats were randomly selected for neurophysiological testing (*n* = 3). Briefly, 0.2% pentobarbital sodium, 50 mg/kg, was injected intraperitoneally, and the rats were fixed in a stereotaxic apparatus until the righting reflex disappeared. The scalp was prepared and sterilized to expose the bregma, and the Schaffer lateral branch (ML = −2.2 mm, AP = −1.2 mm, DV = 1.3 mm) and the granular cell layer in the CA1 area (ML = 1.5 mm, AP = −2.0 mm, DV = 1.5 mm) were located at the origin of the bregma. Drilling was followed by implantation of the electrodes (Kedou Brain-Computer Technology, Suzhou, China). During Phase I, the parameters were adjusted to induce the group peak potential at a frequency of 1/60 Hz, a wave width of 100 s, and a current of 0.3 mA. To obtain the best population spike (PS), the depths of the stimulating and recording electrodes were adjusted. Once the PS stabilized for 30 min, the stimulation intensity was adjusted to 1/3–1/2 of the maximum value, and recording was performed for 30 min as the baseline. In Phase II, five pulses of 400 Hz were repeated three times with a 10-s interval, followed by a 120-min recording of the PS. During the recording, field excitatory postsynaptic potential (fEPSP) slopes were averaged over the last 20 min.

### Western blot analysis

The Whole Cell and Tissue Protein Extraction Kit was used for total protein extraction according to the manufacturer’s protocol (Sigma-Aldrich, Munich, Germany). Protein concentrations were measured using the Bicinchoninic Acid Protein Assay Kit (Thermo Fisher Scientific, Waltham, MA, USA). The hippocampus was removed 1 h after the MWM test (*n* = 3), two tissues were taken from each side of hippocampal CA1 region of each mouse and proteins were separated by 10% sodium dodecyl-sulfate polyacrylamide gel electrophoresis and transferred to polyvinylidene difluoride membranes. The membrane was blocked with 5% skim milk powder for 2 h, and then incubated overnight with primary antibodies (postsynaptic density protein 95 (PSD-95), synaptophysin, MEK, extracellular signal-regulated protein kinase1/2 (ERK1/2), phosphorylated ERK1/2 (p-ERK1/2), cyclic AMP response element binding protein (CREB) and phosphorylated CREB (p-CREB), purchased from Abcam (Cambridge, UK) at a dilution of 1:1,000. Membranes were then rinsed and incubated with secondary antibody for 2 h. A FujiFilm LAS 4000 image analyzer was used to visualize the signals (FujiFilm, Tokyo, Japan).

### Immunofluorescence assay

One day after the MWM test, hippocampal tissues were cut into 20-μm thick slices for triple immunofluorescence staining. The samples were blocked in PBS containing 0.3% Triton X-100 (Sigma-Aldrich, Munich, Germany) and 5% bovine serum album (Beyotime, Beijing, China) for 2 h at room temperature. The samples were then incubated with p-CREB (Abcam, 1:100, Cat No. Ab32096) overnight at 4°C. After washing three times with PBS, the sections were incubated with Cy3-labeled goat anti-rabbit immunoglobulin secondary antibody (1:200, Beyotime, P0186) for 1 h. Finally, the sections were stained with 4′,6-diamidino-2-phenylindole (DAPI; Beyotime, P0131). Two fields of visualization were taken from each hippocampal CA1 region on each side of hippocampus and quantitative analysis was performed using Image Pro Plus 6.0 software.

### Statistical analysis

Statistical analysis was performed using SPSS 21.0 (SPSS, Inc., Chicago, IL, USA). Data are presented as means ± standard deviations. Tukey’s multiple comparison test was performed in addition to one-way analysis of variance for normally distributed data. ANOVA of repeated measurement design and Tukey’s post hoc test were used to analyze escape latency during training. Statistical significance was defined at *p* < 0.05.

### Data availability statement

The data generated during and/or analyzed during the current study are available from the corresponding author on reasonable request.

## RESULTS

### EA prevents cognitive decline induced by anesthesia/surgery in aged rats

**Figure 2:**
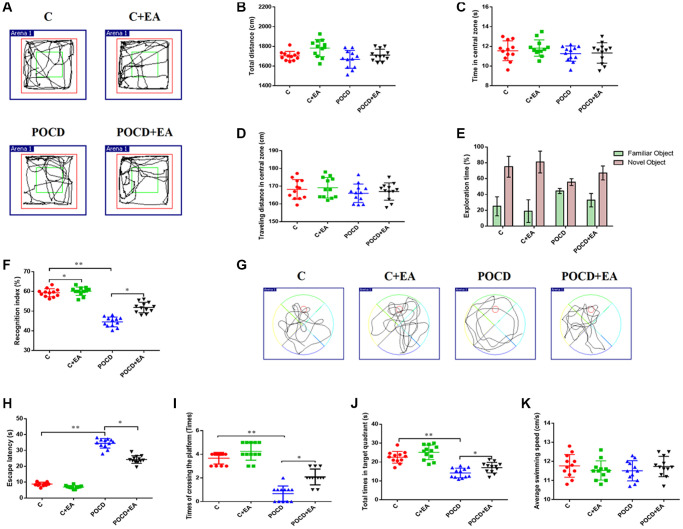
**EA pretreatment alleviates anesthesia and surgery-induced cognitive decline in aged rats.** (**A**) Traveling trajectory in the open field. (**B**–**D**) Total distance traveled, time in the central zone and traveled distance in the central zone of the open field among the four groups. (**E**) Exploration time to the familiar and novel objects during the novel object recognition phase. (**F**) Recognition index. (**G**–**K**) Swimming trajectory, escape latency, number of crossings of the platform area, total time spent in the target quadrant and average swimming speed in the MWM test. ^*^p < 0.05, ^**^p < 0.01.

On the third day following anesthesia/surgery, the OFT was used to examine behavioral changes caused by anesthesia/surgery. There was no significant difference among the four groups in total traveled distance (Figure 2A, 2B), time in the central zone (Figure 2C) or distance in the center (Figure 2A, 2D), showing that surgery and anesthesia do not affect spontaneous locomotor activity in aged rats.

The NORT and MWM tests were used to evaluate learning and memory abilities. Notably, the recognition index was higher in the C and POCD+EA group compared with the POCD group. There was no significant differences between group C and C+EA (Figure 2E, 2F). In the MWM test, the escape latency was shorter and the number of crossings of the platform area and the total time spent in the target quadrant were higher in the C+Y group compared with group C. The escape latency was higher and the number of crossings of the platform area and the total time spent in the target quadrant were lower in the C and POCD+EA group compared with the POCD group. There was no significant differences between C and C+EA group (Figure 2G–2J). There were no significant differences in swimming speed among the four groups (Figure 2K).

### EA pretreatment alters the gene expression profile in the hippocampal CA1 region in aged rats with POCD

**Figure 3:**
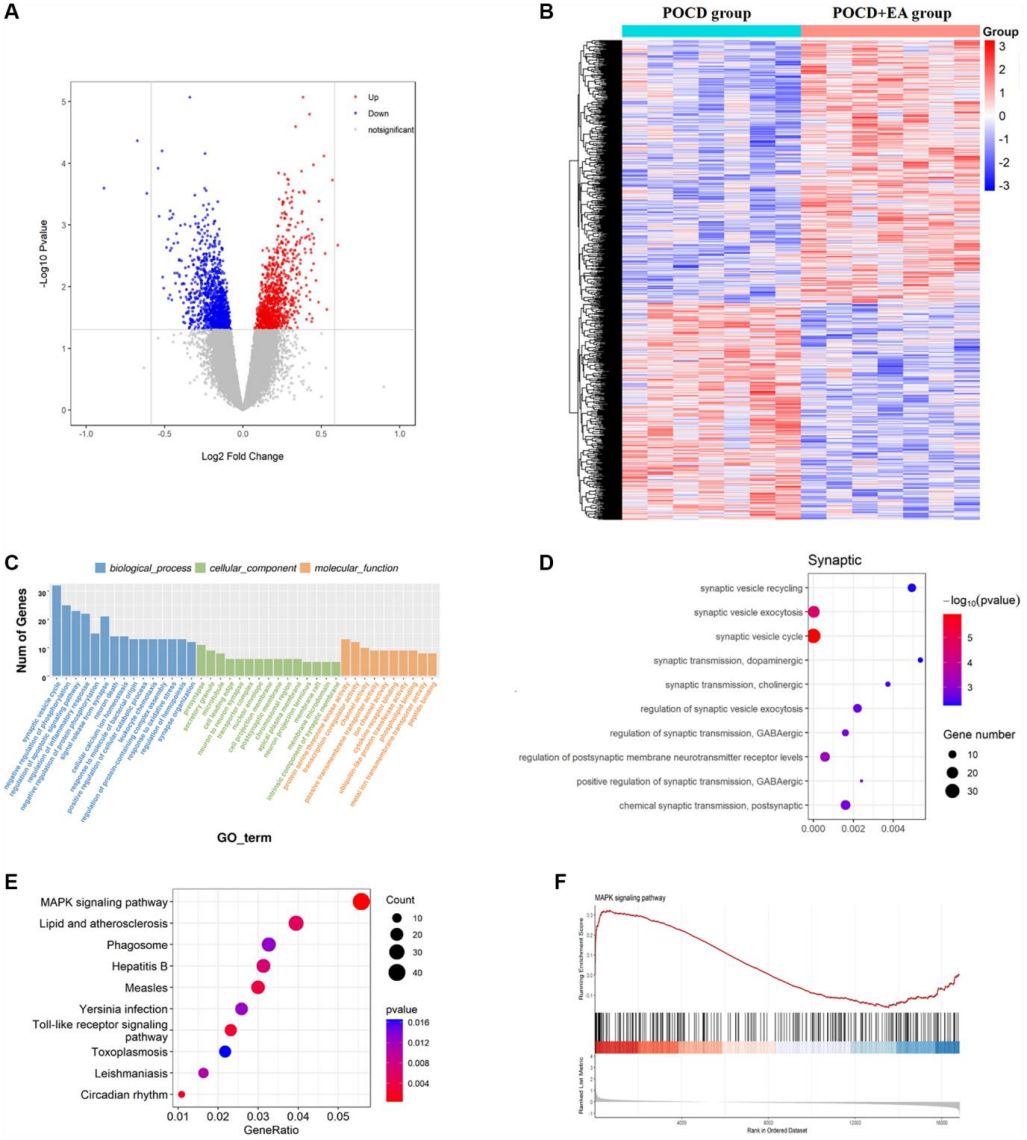
**EA pretreatment alters the gene expression profile in the hippocampal CA1 region of POCD aged rats.** (**A**) Differential gene volcano map. (**B**) Heatmap of differential gene cluster analysis. (**C**) GO enrichment analysis pathways. (**D**) GO enrichment analysis of synaptic pathways. (**E**) KEGG pathway enrichment scatter diagram. (**F**) GSEA gene enrichment analysis diagram of the MAPK signaling pathway.

As a first step towards revealing whether EA pretreatment plays a role in aged rats with POCD, we examined differences in RNA-seq gene expression profiles in the hippocampal CA1 region between the POCD and POCD+EA group. Among the DEGs, 353 were upregulated and 563 were downregulated. DEG volcano maps were created using the ggplot2 package in R software (Figure 3A), and DEG cluster analysis heatmaps were drawn using the R software package, pheatmap (Figure 3B). In accordance with the GO pathway diagram, DEGs were enriched for “regulation of membrane potential” “neuron to neuron synapse” and “protein serine/threonine kinase activity” (Figure 3C) and synapse-related GO terms such as synaptic vesicle recycling, synaptic vesicle exocytosis and synaptic vesicle cycle were significantly enriched (Figure 3D). KEGG pathway analysis indicated significant enrichment of 330 signaling pathways. According to the KEGG pathway map, “MAPK signaling pathway”, “Lipid and atherosclerosis”, “Phagosome”, “Hepatitis” and “Measles” were the top five enriched pathways (Figure 3E). Additionally, GSEA revealed significant enrichment of the MAPK signaling pathway (enrichment score: 0.62, *p* = 0.002; Figure 3F).

### EA pretreatment ameliorates synaptic plasticity in the hippocampal CA1 region in aged rats with POCD

**Figure 4:**
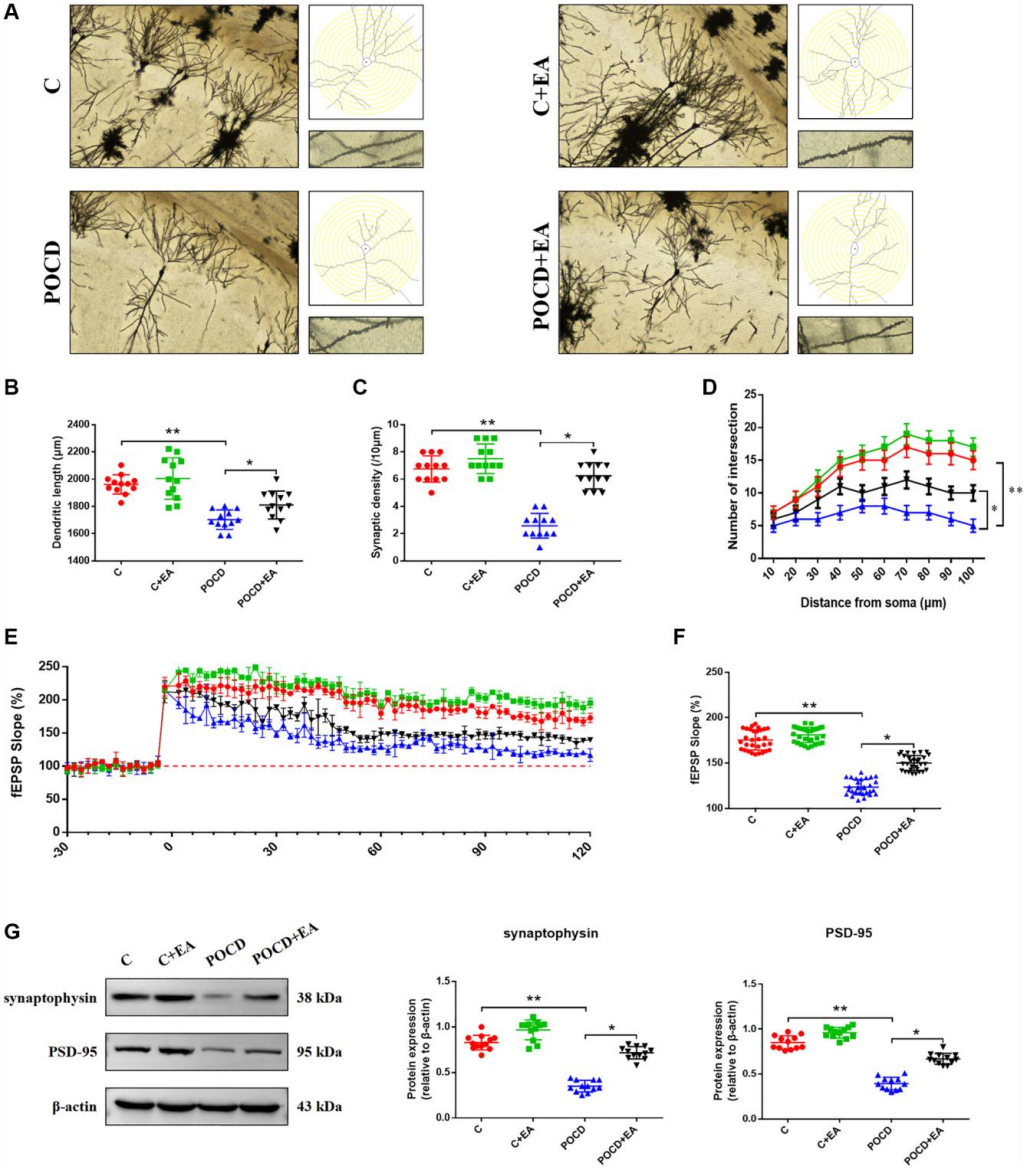
**EA pretreatment ameliorates the anesthesia/surgery-induced impairment in synaptic plasticity in the hippocampal CA1 region in aged rats.** (**A**) Representative hippocampal Golgi-Cox staining images showing dendritic arborization in the hippocampal CA1 region (scale bar: 100 μm or 20 μm for enlarged inserts) and diagram of dendritic Sholl analysis. (**B**–**D**) Quantitative analysis of total synaptic length, synaptic spine density and the number of intersections of dendrites in the CA1 region. (**E**) Analysis of local field potentials in the CA1 region in response to stimulation of Schafer lateral branch fibers. (**F**) Average fEPSP slope in the last 20 min (*n* = 3). (**G**) The expression of synaptophysin and PSD-95. Data are shown as means ± standard deviations. ^*^p < 0.05, ^**^p < 0.01.

To further investigate memory function, we stained the hippocampal CA1 region by Golgi staining to observe changes in synaptic structure (Figure 4A). The dendritic length (Figure 4B), synaptic spine density (Figure 4C), number of intersections of dendrites (Figure 4D), LTP (Figure 4E, 4F) and expression level of synaptophysin and PSD-95 (Figure 4G) in the CA1 region were higher in C group compared with the POCD group. Notably, EA pretreatment prevented the reductions in dendritic length (Figure 4B), synaptic spine density (Figure 4C), number of intersections of dendrites (Figure 4D), LTP (Figure 4E, 4F) and expression level of synaptophysin and PSD-95 (Figure 4G) in the POCD+EA group. There was no significant differences between group C and C+EA.

### EA pretreatment activates CREB via the ERK/MAPK pathway to exert a neuroprotective effect in rats with POCD

**Figure 5:**
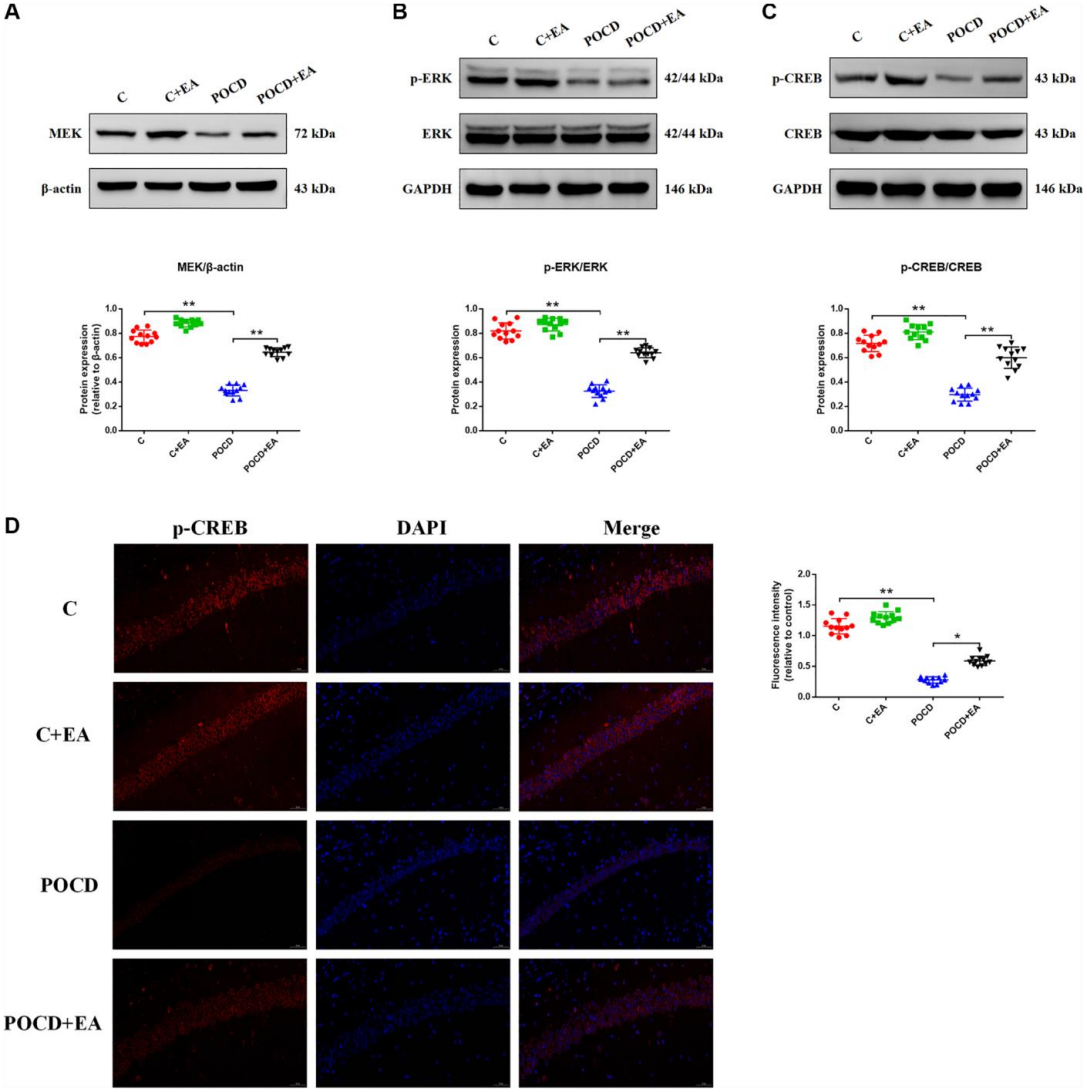
**EA pretreatment activates the MAPK/ERK/CREB signaling pathway to exert a neuroprotective effect in rats with POCD.** The expression of proteins related to the MAPK/ERK/CREB signaling pathway, including MEK (**A**), phosphorylated ERK/ERK (**B**) and phosphorylated CREB (pCREB)/CREB (**C**). (**D**) Representative immunostaining showing the colocalization of p-CREB (red) and DAPI (blue) in the CA1 of the hippocampus. Scale bar: 200 μm. Data are shown as means ± standard deviations. ^*^p < 0.05, ^**^p < 0.01.

CREB plays a key role in improving synaptic plasticity by activating the MAPK/ERK signaling pathway [27]. Thus, we hypothesized that it might also alleviate POCD in aged rats following pretreatment with EA. To test this, we analyzed the expression of MEK, ERK, p-ERK, CREB and p-CREB by western blotting. The expression levels of MEK (Figure 5A), p-ERK (Figure 5B) and p-CREB (Figure 5C) were significantly downregulated in the hippocampus of rats in the POCD group compared with the C group. The expression levels of MEK, p-ERK and p-CREB were upregulated in the POCD+EA group compared with the POCD group (Figure 5A–5C). However, the expression levels of ERK and CREB did not differ among the four groups. There was no significant differences between group C and C+EA (Figure 5A–5C). Immunostaining for p-CREB, which is downstream of the MAPK/ERK/CREB signaling pathway, revealed that the intranuclear expression of the transcription factor in the hippocampus was lower in the POCD group compared with the C group, whereas it was higher in the POCD+EA group than in the POCD group. There was no significant differences between group C and C+EA (Figure 5D).

### Inhibition of the MAPK/ERK/CREB pathway in the CA1 region blocks the neuroprotective effect of EA

**Figure 6:**
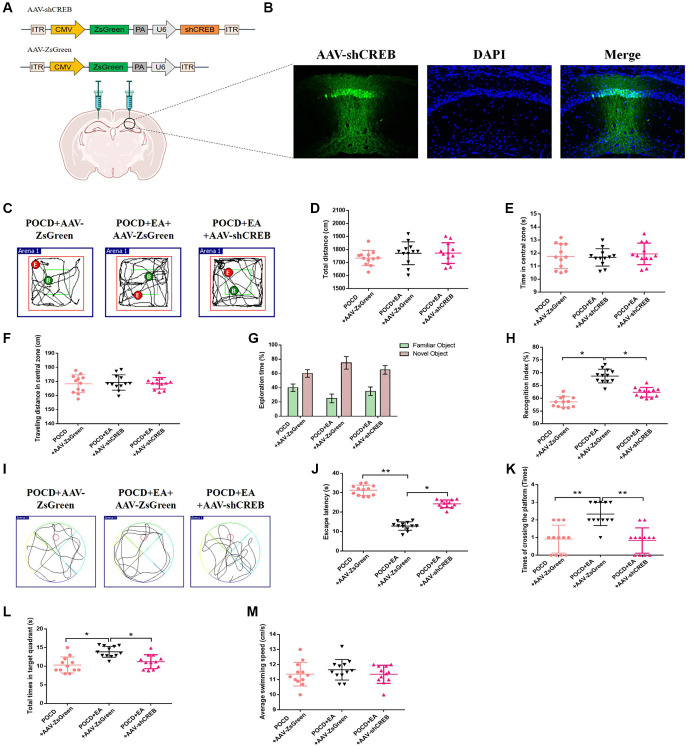
**Inhibition of the MAPK/ERK/CREB pathway in the CA1 region reduces the protective effect of EA against postoperative cognitive dysfunction.** (**A**) The CA1 was bilaterally injected with AAV-shCREB 21 days before surgery. (**B**) ZsGreen was specifically expressed in hippocampal CA1 region. Scale bar = 50 μm. (**C**) Traveling trajectory in the open field. (**D**–**F**) Total distance traveled, time in the central zone and traveled distance in the central zone of the open field in the three groups. (**G**) Exploration time to the familiar and novel objects during the novel object recognition phase. (**H**) Recognition index. (**I**–**M**) Swimming trajectory, escape latency, number of crossings of the platform area, total time spent in the target quadrant and average swimming speed in the MWM test. Data are shown as means ± standard deviations. ^*^p < 0.05, ^**^p < 0.01.

To investigate the role of the MAPK/ERK/CREB pathway in neuroprotection of EA pretreatment, we injected AAV-shCREB into the bilateral hippocampal CA1 region of aged rats 21 days before surgery to downregulate CREB (Figure 6A, 6B), as described in the previous study [28]. There were no differences in locomotor activity among the three groups (*p* > 0.05; Figure 6C–6F). Additionally, the recognition index was higher in the POCD+AAV-ZsGreen+EA group compared with the POCD+AAV-ZsGreen and POCD+AAV-ZsGreen+EA groups (Figure 6G, 6H). In the MWM test, the escape latency was longer and the number of crossings of the platform area and the total time spent in the target quadrant were lower in the POCD+AAV-ZsGreen+EA group compared with the POCD+AAV-ZsGreen and POCD+AAV-ZsGreen+EA groups (Figure 6I–6M).

**Figure 7:**
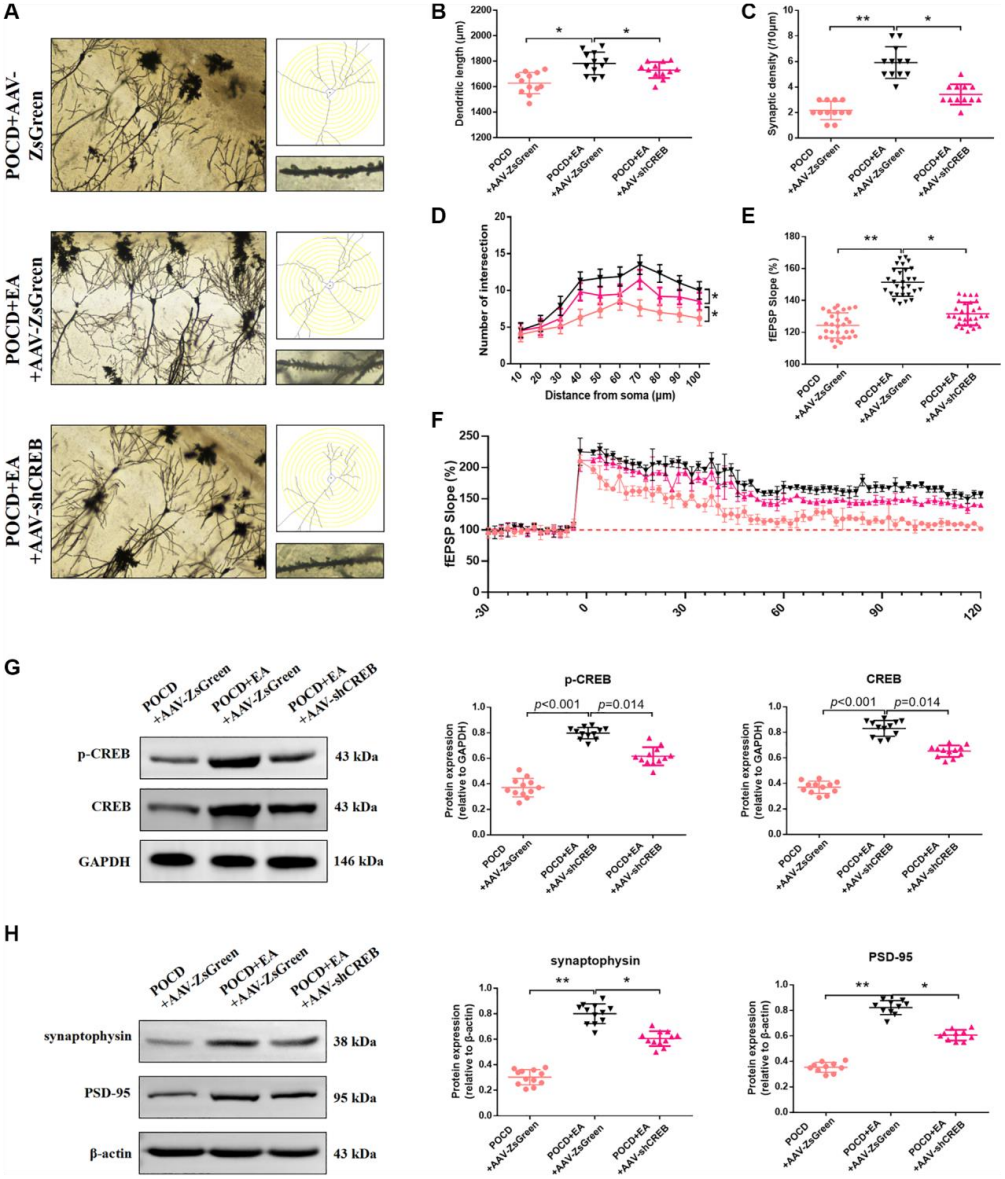
**Inhibition of the MAPK/ERK/CREB pathway in the CA1 region reduces the ability of EA to improve synaptic plasticity.** (**A**) Representative hippocampal Golgi-Cox staining images showing dendritic arborization in hippocampal CA1 pyramidal neurons (scale bar: 100 μm, or 20 μm for enlarged inserts) and diagram of dendritic Sholl analysis. (**B**–**D**) Quantitative analysis of total synaptic length, synaptic spine density and the number of intersections of dendrites (analyzed by repeated measures *t*-test, with corrected *p*-values) in CA1 pyramidal neurons. (**E**) Average fEPSP slope in the last 20 min. (**F**) Analysis of local field potentials in the CA1 region in response to stimulation of Schafer lateral branch fibers. The expression of p-CREB and CREB (**G**). The expression of synaptophysin and PSD-95 (**H**). Data are shown as means ± standard deviations. ^*^p < 0.05, ^**^p < 0.01.

Western blotting and Golgi staining were used to evaluate synaptic plasticity in the hippocampal CA1 region in aged rats. The dendritic length (Figure 7A, 7B), synaptic density (Figure 7A, 7C), number of intersections (Figure 7A, 7D), LTP (Figure 7E, 7F), expression level of synaptophysin, PSD-95 and p-CREB (Figure 7G, 7H) were increased in the POCD+AAV-ZsGreen+EA group and compared with the POCD+AAV-ZsGreen POCD+AAV-ZsGreen+EA groups.

## DISCUSSION

**Figure 8:**
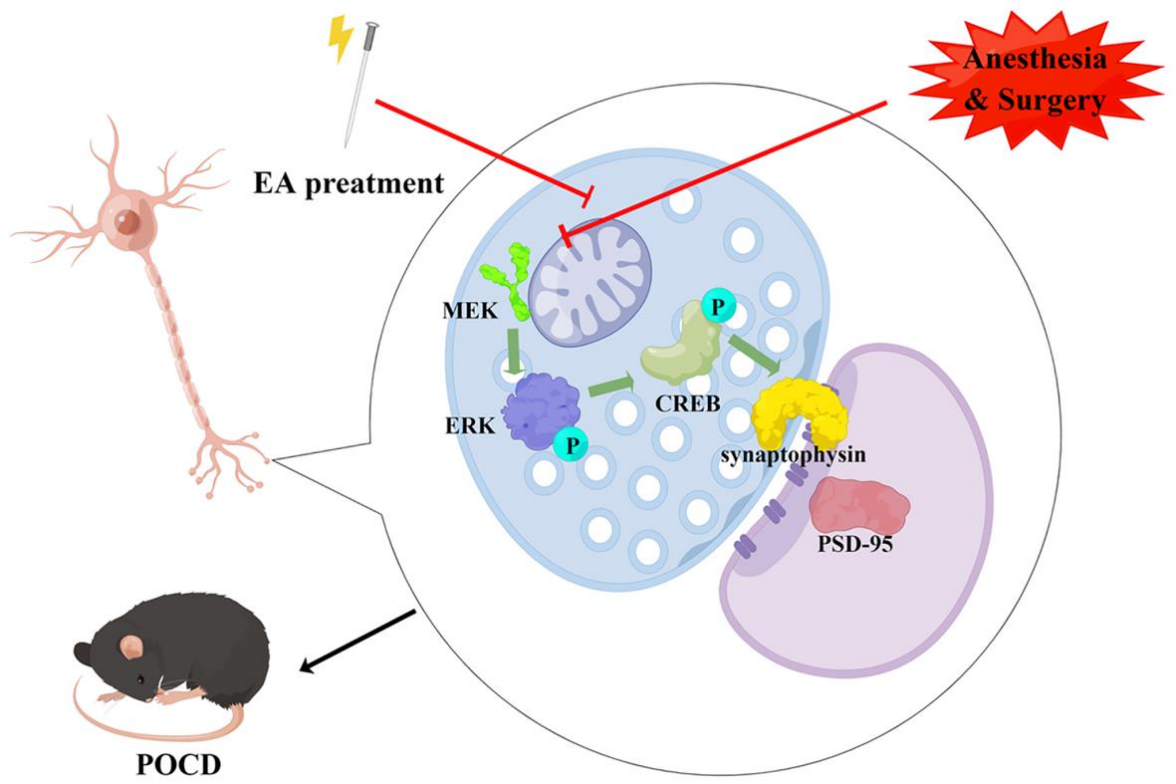
**Schematic diagram of the neuroprotective effect of EA pretreatment (drawn by Figdraw, ID: IWWRSae558).** EA pretreatment improves synaptic plasticity by activating CREB via the ERK/MAPK pathway in the hippocampal CA1 region, thereby inhibiting cognitive decline caused by surgery and anesthesia in aged rats.

Here, we investigated the mechanisms underlying the neuroprotective effect of EA on anesthesia/surgery-induced cognitive decline in aged rats. We found that EA pretreatment improved recognition memory, prevented memory impairments and enhanced learning ability in aged rats with POCD, consistent with our previous study [25]. Furthermore, EA improved synaptic plasticity in the hippocampal CA1 region and activated the MAPK/ERK/CREB pathway in aged rats with POCD. Our findings suggest that pretreatment with EA may be a valuable therapeutic target for preventing anesthesia/surgery-induced cognitive decline (Figure 8).

As a burgeoning treatment method, EA, based on acupuncture and moxibustion, energizes the needle at different frequencies and intensities, and has become a complementary therapy [29–31]. EA may also have neuroprotective effects in addition to its well-known pain management properties [32, 33]. Xie L showed EA improves recognition abilities and memory by inhibiting NF-κB pathway but activating Stat6 pathway and improving immunity of AD rats [34]. Moreover, in certain brain areas, EA enhances autophagy initiation, autophagosome biogenesis, mitophagy, and autophagic flux/substrate degradation, which has exciting therapeutic potential [35]. Hegu is a major acupoint on the Yangming Meridian of the large intestine of the hand. It calms pain, dries meridians and activates collaterals. In addition to calming static pain, dredging meridians and activating meridians, Neiguan point is among the most widely used acupoints on the hand Jueyin pericardial meridian. Zusanli situated along the “zuyangming Stomach Meridian,” holds significant prominence as a primary acupoint. Its robust efficacy renders it an important point pivotal locus for health care interventions. Our previous research found that pretreatment with EA at Neiguan, Hegu and Zusanli improved anesthesia and surgery-induced POCD in aged rats by inhibiting HMGB1-NF-κB via an α7-nAChR signal in microglia [16] or ameliorating mitochondrial damage and neuroapoptosis [15]. However, the neuroprotective mechanism of EA has not yet been fully elucidated.

Postoperative complications in elderly patients not only prolong hospitalization and increase treatment costs, but also increase the risk of other complications. To prevent POCD in older adults, effective measures must be found. In this study, to simulate anesthetic surgical procedures in clinically aged patients, we performed exploratory laparotomy under sevoflurane anesthesia on 20-month-old rats. On day 3 after anesthesia/surgery, The OFT was performed to assess spontaneous locomotor activity. The exploratory laparotomy and the postoperative analgesia did not impact locomotor activity in the aged rats. The MWM and NOR tests demonstrated cognitive impairment in the aged rats after anesthesia/surgery, demonstrating successful establishment of the POCD model. Notably, EA pretreatment didn’t affect cognitive function in aged rats without surgery, and postoperatively, EA pretreatment significantly improved cognitive function in aged rats, consistent with our previous research findings [15].

The hippocampus plays major roles in learning and memory and emotional regulation [36, 37]. Synaptic plasticity refers to changes in synaptic efficacy over time, which is crucial to learning and memory [37]. Hippocampal synaptic plasticity disorder is involved in many pathological states and the occurrence and development of neurodegenerative diseases, while synaptic plasticity plays an important role in the treatment of neurodegenerative diseases and participates in brain network reconstruction following brain injury [38]. There is a strong correlation between synaptic plasticity and cognitive dysfunction. Elderly patients show an increased central inflammatory response after surgery, and abnormal expression of inflammatory mediators impairs synaptic plasticity, leading to cognitive dysfunction and anxiety and depression [39–41]. Proteomic analysis showed that the expression of synaptic plasticity-related proteins (dynamin-1 and dihydropyrimidinase-related protein 2) are significantly reduced in the aging perioperative neurocognitive disorder mouse model [42]. In addition, POCD in elderly rats having undergone laparotomy under anesthesia is related to a reduction in the synaptic plasticity-related protein PSD95 and the range of synaptic active regions and synaptic curvature in the hippocampus [43]. In the current study, we performed RNA-seq analysis to identify DEGs associated with EA-induced neuroprotection in aged rats with POCD. GO analysis revealed that “neuron to neuron synapse” and synapse-related GO terms such as synaptic vesicle recycling, synaptic vesicle exocytosis and synaptic vesicle cycle were significantly enriched. We used Golgi-Cox staining to evaluate synaptic morphology and LPT recordings and synaptophysin and PSD-95 immunostaining to assess changes in synaptic plasticity. Pretreatment with EA significantly improved synaptic plasticity in aged rats with POCD, consistent with our previous research results.

In this study, the RNA-seq analysis showed that “MAPK signaling pathway” was significantly enriched by KEGG analysis. Mitogen-activated protein kinase (MAPK) is a serine/threonine protein kinase that is a key downstream effector in Ras signaling. There are four parallel signal transduction pathways within the MAPK family, and the ERK1/2 signaling pathway is the first to have been discovered and plays an important role in the regulation of synaptic plasticity [44, 45]. Upon activation of the MAPK/ERK pathway, ERK1/2 is phosphorylated. CREB is a key transcription factor that regulates neurogenesis and neural plasticity-related genes, and plays a key role in long-term learning and memory. Phosphorylated ERK1/2 can further phosphorylate CREB, thereby regulating synaptic plasticity and cognitive functions [46].

To gain mechanistic insight, we generated AAVs to downregulate CREB. Compared with the POCD+AAV-ZsGreen+EA group, cognitive function was significantly declined in the POCD+EA+AAV-shCREB group. Golgi staining and neurophysiological recordings were then used to investigate structural and functional synaptic plasticity. The density and number of dendritic spines and the amplitude of LTP in the POCD+EA+AAV-shCREB group were significantly decreased. These results indicate that the EA improves anesthesia and surgery-induced cognitive impairment through activation of the MAPK/ERK/CREB pathway in hippocampal neurons improves synaptic function.

There are, however, some limitations to this study. First, we only examined memory function within 7 days postoperatively in aged rats without testing long-term memory. Second, we only performed EA stimulation on Baihu, Dazhui and Zusanli acupoints, and no other acupoints were examined. Thirdly, we only explored the impact of preoperative EA intervention on POCD. Whether postoperative EA intervention will achieve the same effect will be a more practical and interesting topic in clinical practice, and we will also explore it in future studies. Fourthly, we only conducted in-depth exploration on the role of synaptic plasticity and MAPK signaling pathway in the improvement of POCD by EA pretreatment based on the results of RNA sequencing, “regulation of apoptotic signaling pathway” and “regulation of inflammation response” may also be potential intervention targets for electroacupuncture, which is also a problem that we need to pay attention to in the future. Finally, other POCD models, such as tibial fracture, splenectomy or partial hepatectomy, should also be used to investigate the protective effect of EA in future studies.

## CONCLUSION

Our findings show that anesthesia/surgery in aged rats leads to hippocampal-dependent cognitive dysfunction. Impaired synaptic plasticity is an important mechanism in POCD. EA pretreatment alleviates anesthesia/surgery-induced cognitive decline in aged rats by ameliorating synaptic plasticity via activation of the MAPK/ERK/CREB pathway. EA pretreatment may therefore have clinical potential in the prevention of POCD.
